# Supplementary material for: HDAC11 Regulates RNA Splicing via De-Fatty Acylation of SF3B2
Source: bioRxiv. 2026 Feb 23:2026.02.22.707301. Preprint. [Version 1] doi: 10.64898/2026.02.22.707301 (PMC13160072; doi:10.64898/2026.02.22.707301)
Supplement: Supplement 2 [file NIHPP2026.02.22.707301v1-supplement-2.pdf]

bioRxiv preprint doi: https://doi.org/10.64898/2026.02.22.707301; this version posted February 23, 2026. The copyright holder for this preprint (which was not certified by peer review) is the author/funder, who has granted bioRxiv a license to display the preprint in perpetuity. It is made available under aCC-BY 4.0 International license.

Figure S1. HDAC11 and SF3B2 expression in HCC patients. (A) HDAC11 survival plot in HCC patients**. (B)** SF3B2 survival plot in HCC patients. **(C)** Survival plot analyzing combined expression of HDAC11 and SF3B2 in HCC patients. (**D-E)** Normal versus tumor expression of HDAC11 and SF3B2 from HCC patients. Statistics are by Mann Whitney U-test. ****p<0.0001.

Figure S2. HDAC11 loss of function mutants are structurally similar to WT HDAC11 but disrupt the interaction with SF3B2. (A) Co-immunopurification of WT HDAC11 versus HDAC11 mutants with SF3B2**. (B)** Alpha Fold 3 predication of HDAC11 structure. **(C)** Overlay of two separate predictions of HDAC11 WT structure. **(D)** Overlay of HDAC11 WT and HDAC11 D181A structure. **(E)** Overlay of HDAC11 WT and HDAC11 H183A structure. **(F)** Overlay of HDAC11 WT and HDAC11 Y304H structure **(G)** RMSD and Sequence Alignment Scores of HDAC11 structures.

Figure S3. HDAC11 de-fatty acylates endogenous SF3B2 in the nucleus but does not deacetylate Histone H3. (A) Endogenous SF3B2 fatty acylation affinity purification in HDAC11 KD cells with overexpressed WT and Y304H HDAC11**. (B)** Quantification of H **(C).** Hep-G2 cell fractionation to assess HDAC11 and SF3B2 localization. **(D)** Localization of SF3B2 between Hep-G2 parental (par), pLKO.1 control (ctrl), or HDAC11 knockdown (KD) stable cell lines**. (E)** Western blot of H3 acetylation marks from Hep-G2 nuclear fraction.

Figure S4. SF3B2 SUMOylation at K10 is modulated by fatty acylation. (A) SF3B2 can be SUMOylated by SUMO1. This is a representative image from 2 independent experiments. **(B)** Top 10 predicted SUMOylation sites on SF3B2. **(C)** K10 SUMO motif. **(D)** SF3B2 WT versus K10R SUMOylation. Image is representative of 2 independent experiments. **(E)** Analysis of

bioRxiv preprint doi: https://doi.org/10.64898/2026.02.22.707301; this version posted February 23, 2026. The copyright holder for this preprint (which was not certified by peer review) is the author/funder, who has granted bioRxiv a license to display the preprint in perpetuity. It is made available under aCC-BY 4.0 International license.

SF3B2 SUMOylation versus Alk14 labelling**.** **(F)** Analysis of SF3B2 SUMOylation with and without HDAC11 overexpression.

Figure S5: Comparison of AR-FL and AR-v7 expression in PCa and HCC cells. (A) Western blot comparison between 22Rv1, PC3, Hep-G2, and Huh-7 cells. PC3 cells are the negative control. **(B)** qPCR comparison of AR-FL, normalized against Hep-G2 expression level. **(C)** qPCR comparison of AR-v7, normalized against Hep-G2 expression level. **(D)** Comparison of the AR-v7/FL ratio across cell lines.

Each shape in dot plots represents independent experiments. ns: not significant by ANOVA.

Figure S6: Huh-7 cells AR alternative splicing is regulated by HDAC11. (A) Western blot confirming overexpression of HDAC11-HA. **(B)** qPCR analysis of AR exon junctions after HDAC11 overexpression**. (C)** Relative AR-v7/FL ratio after HDAC11 overexpression**. (D)** AR-v7/FL splicing ratio with WT or K10R SF3B2**. (E)** Western blot confirming KD of HDAC11**. (F)** qPCR analysis of AR exon junctions after HDAC11 KD**. (G)** Relative AR-v7/FL ratio after HDAC11 KD**.**

Each shape in dot plots represents independent experiments. $*p<0.05$, $**p<0.01$, $***p<0.001$, ns: not significant by t-test

bioRxiv preprint doi: https://doi.org/10.64898/2026.02.22.707301; this version posted February 23, 2026. The copyright holder for this preprint (which was not certified by peer review) is the author/funder, who has granted bioRxiv a license to display the preprint in perpetuity. It is made available under aCC-BY 4.0 International license.

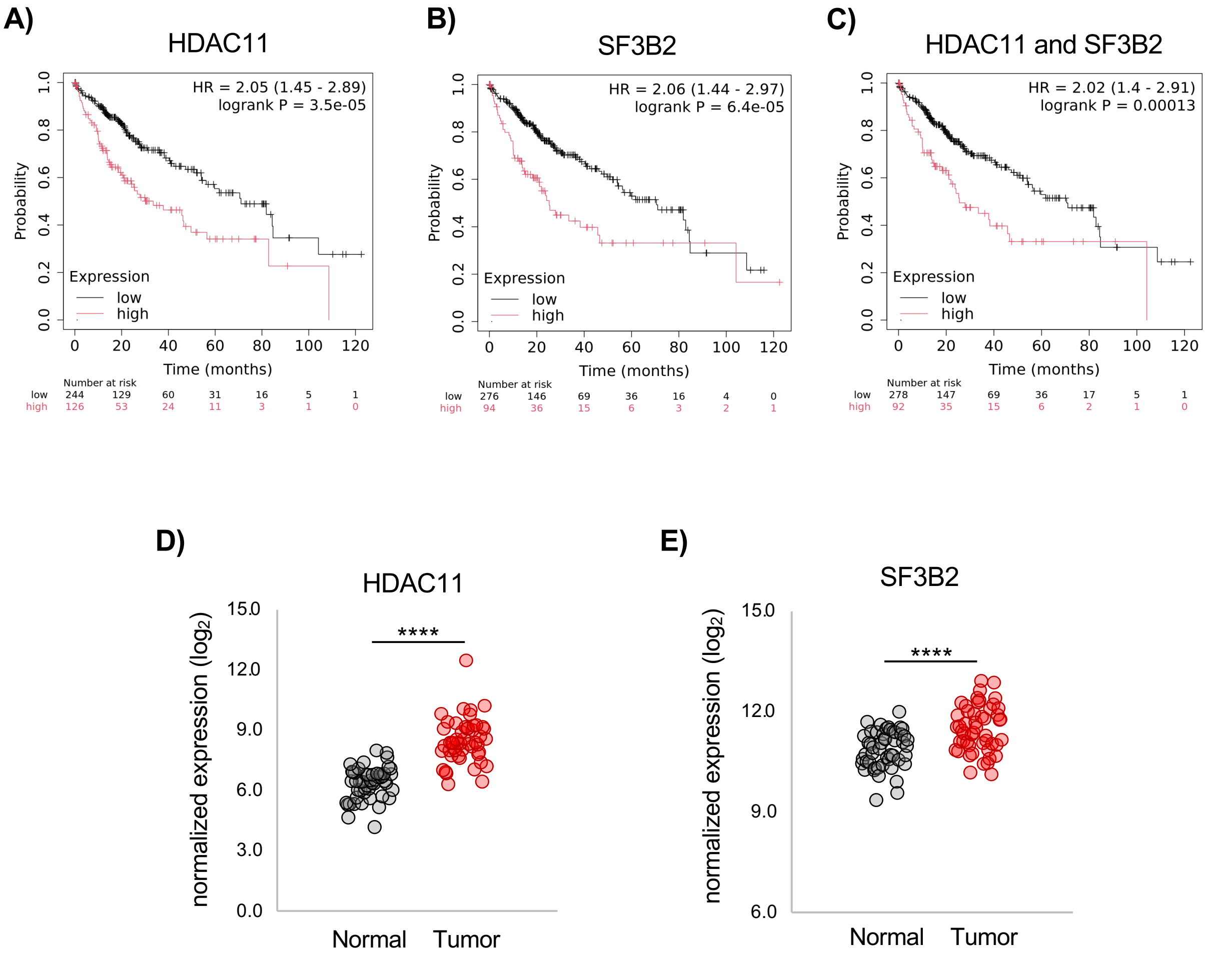

Clements, et al. Supplementary Figure 1.

bioRxiv preprint doi: https://doi.org/10.64898/2026.02.22.707301; this version posted February 23, 2026. The copyright holder for this preprint (which was not certified by peer review) is the author/funder, who has granted bioRxiv a license to display the preprint in perpetuity. It is made available under aCC-BY 4.0 International license.

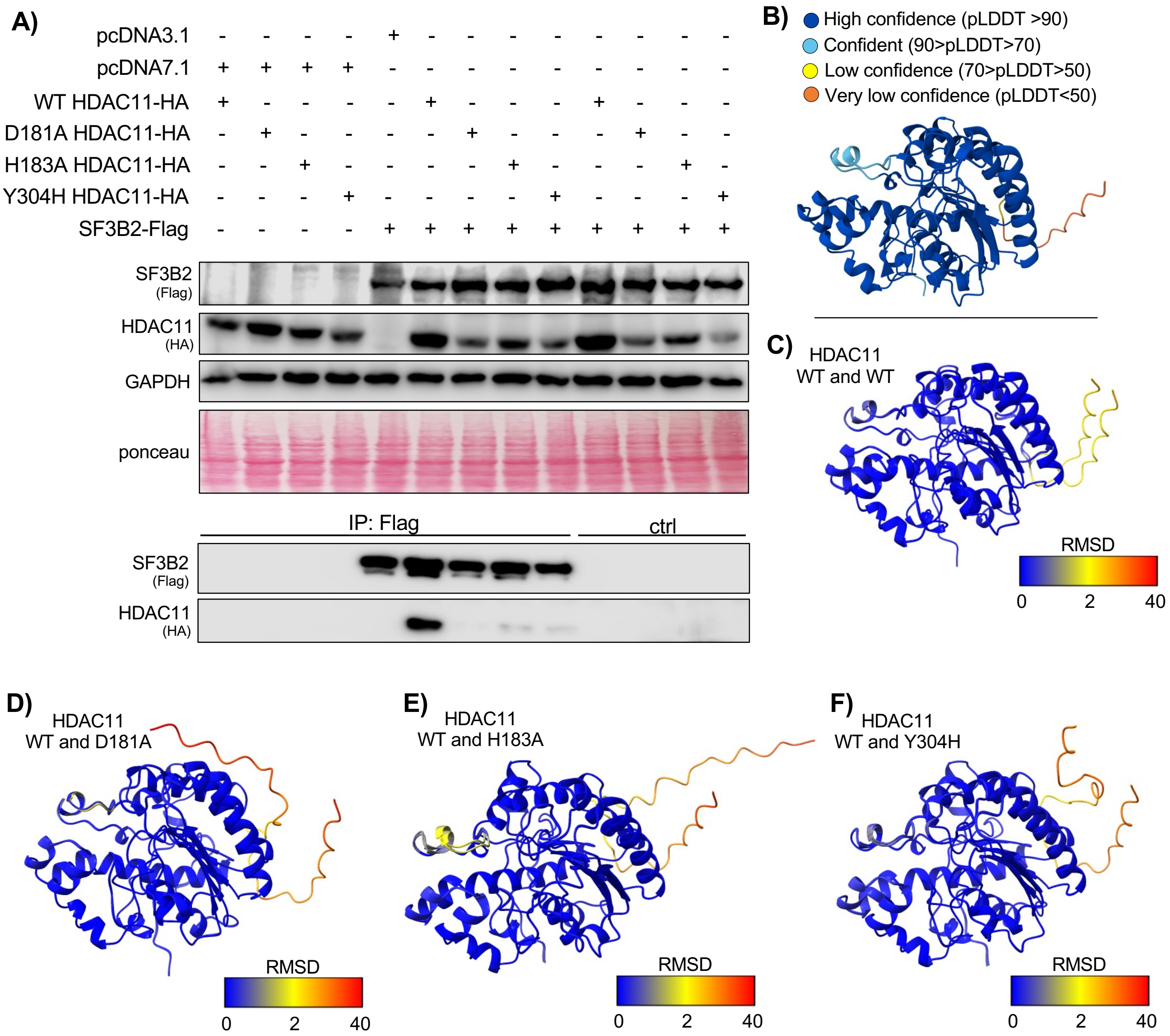

G)

WT HDAC11 paired with:	RMSD (without outlier regions)	RMSD (with outliers)	Sequence Alignment Score
**WT**	0.135	1.211	1791.9
**D181A**	0.138	6.457	1783.5
**H183A**	0.213	5.038	1774.9
**Y304H**	0.177	4.882	1785.6

Clements, et al. Supplementary Figure 2.

bioRxiv preprint doi: https://doi.org/10.64898/2026.02.22.707301; this version posted February 23, 2026. The copyright holder for this preprint (which was not certified by peer review) is the author/funder, who has granted bioRxiv a license to display the preprint in perpetuity. It is made available under aCC-BY 4.0 International license.

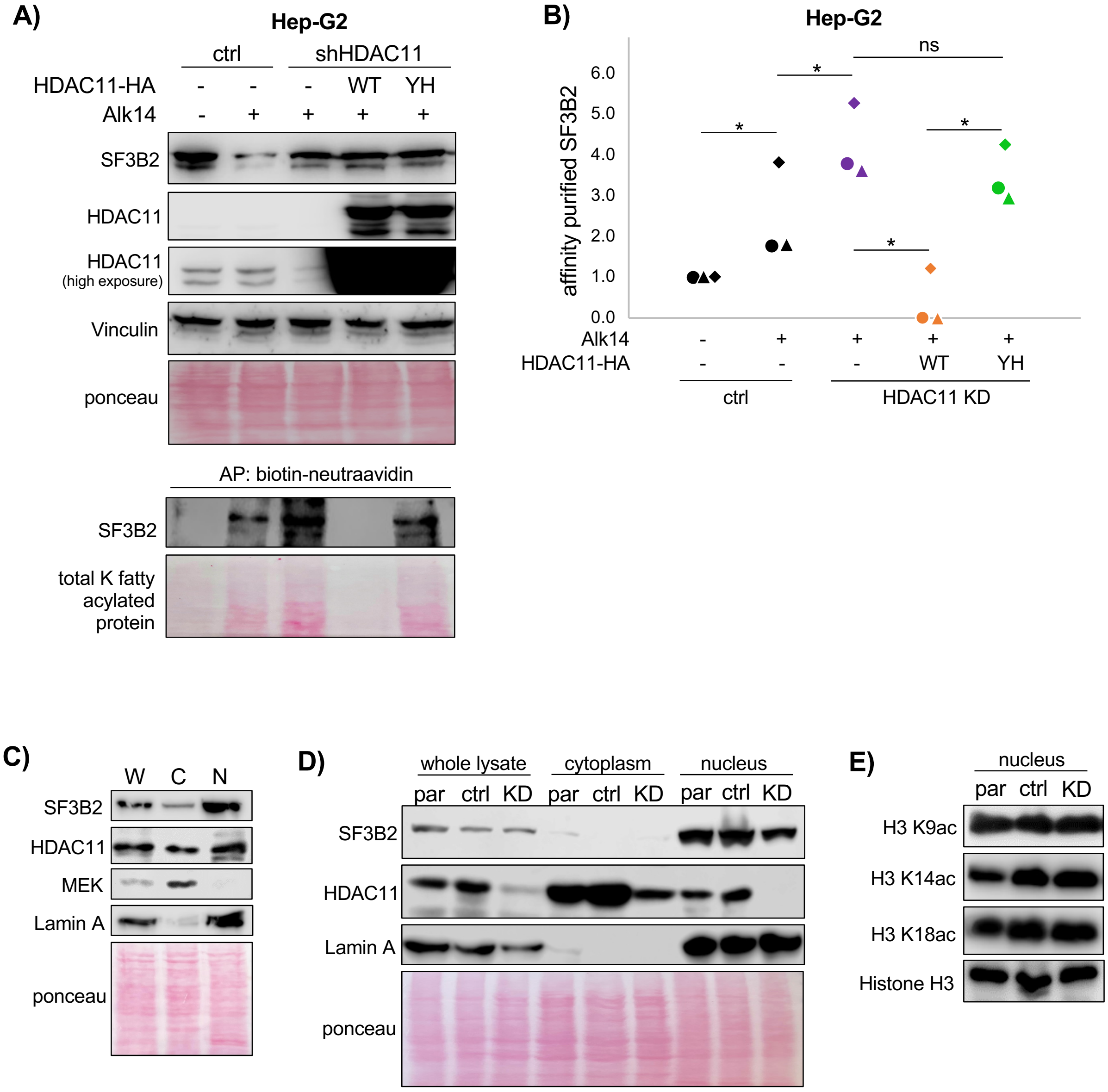

Clements, et al. Supplementary Figure 3.

bioRxiv preprint doi: https://doi.org/10.64898/2026.02.22.707301; this version posted February 23, 2026. The copyright holder for this preprint (which was not certified by peer review) is the author/funder, who has granted bioRxiv a license to display the preprint in perpetuity. It is made available under aCC-BY 4.0 International license.

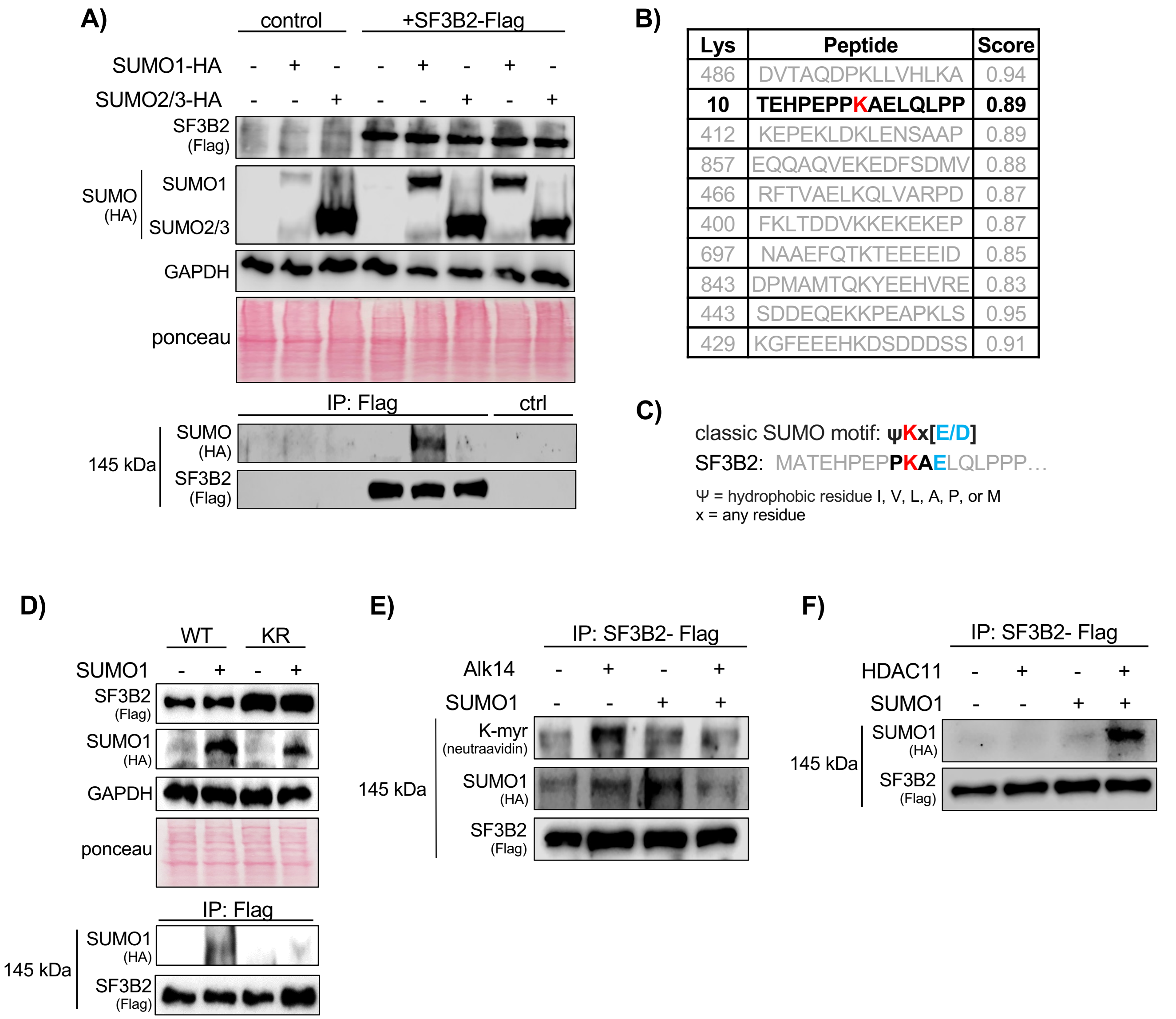

Lys	Peptide	Score
486	DVTAQDPKLLVHLKA	0.94
**10**	**TEHPEPPKAELQLPP**	**0.89**
412	KEPEKLDKLENSAAP	0.89
857	EQQAQVEKEDFSDMV	0.88
466	RFTVAELKQLVARPD	0.87
400	FKLTDDVKKEKEKEP	0.87
697	NAAEFQTKTEEEEID	0.85
843	DPMAMTQKYEEHVRE	0.83
443	SDDEQEKKPEAPKLS	0.95
429	KGFEEEHKDSDDDSS	0.91

Clements, et al. Supplementary Figure 4.

bioRxiv preprint doi: https://doi.org/10.64898/2026.02.22.707301; this version posted February 23, 2026. The copyright holder for this preprint (which was not certified by peer review) is the author/funder, who has granted bioRxiv a license to display the preprint in perpetuity. It is made available under aCC-BY 4.0 International license.

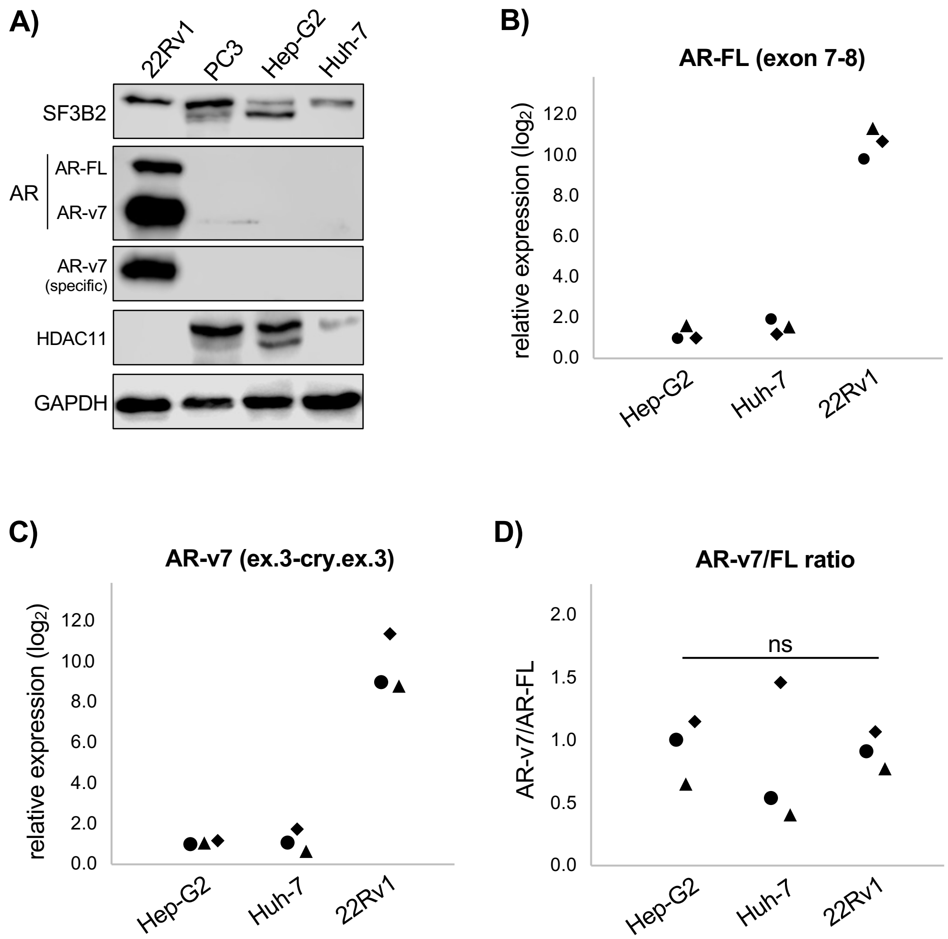

Clements, et al. Supplementary Figure 5.

bioRxiv preprint doi: https://doi.org/10.64898/2026.02.22.707301; this version posted February 23, 2026. The copyright holder for this preprint (which was not certified by peer review) is the author/funder, who has granted bioRxiv a license to display the preprint in perpetuity. It is made available under aCC-BY 4.0 International license.

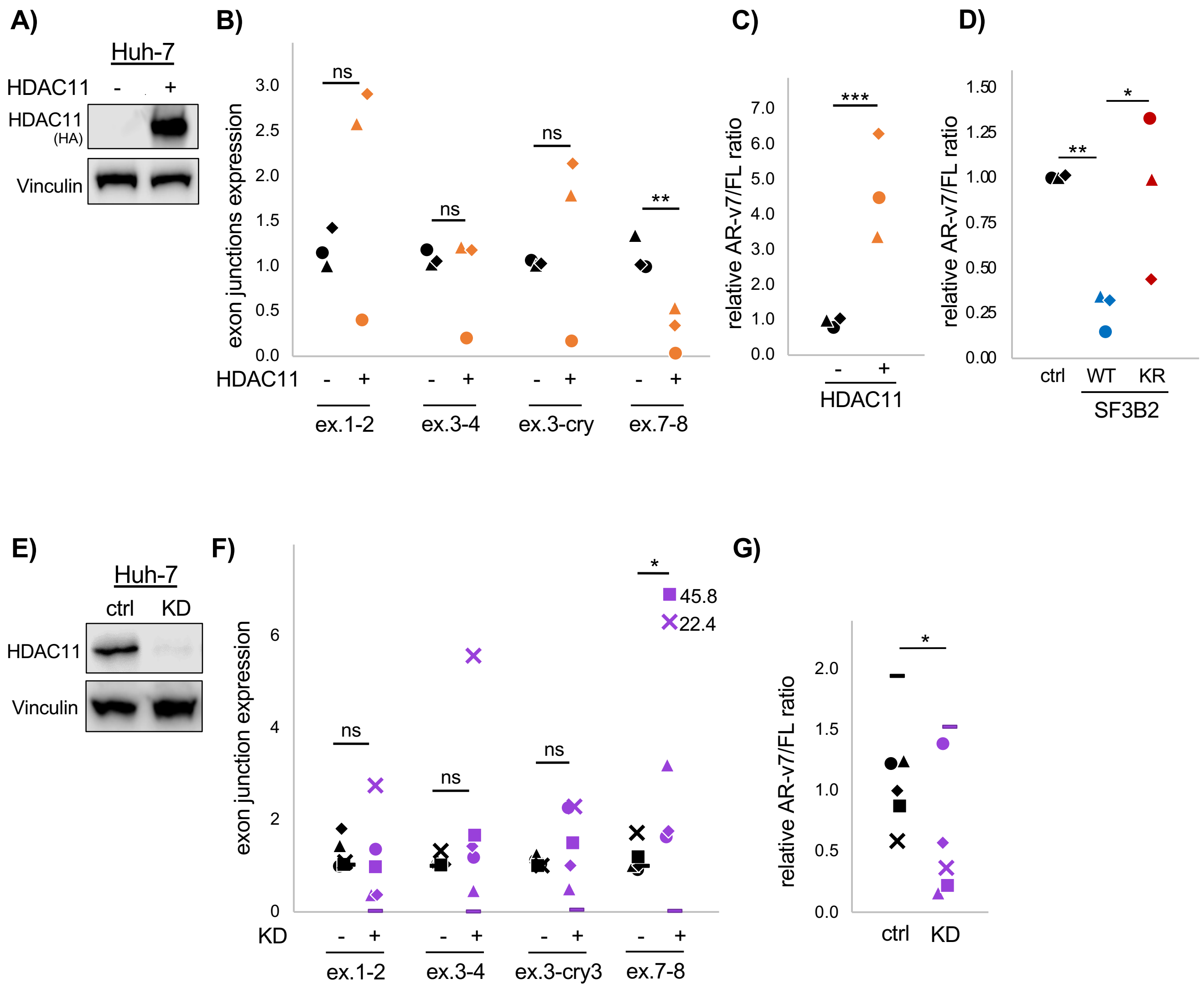

Clements, et al. Supplementary Figure 6.
